# Beta-lactamase database (BLDB) – structure and function

**DOI:** 10.1080/14756366.2017.1344235

**Published:** 2017-07-19

**Authors:** Thierry Naas, Saoussen Oueslati, Rémy A. Bonnin, Maria Laura Dabos, Agustin Zavala, Laurent Dortet, Pascal Retailleau, Bogdan I. Iorga

**Affiliations:** a Service de Bactériologie-Hygiène, Hôpital de Bicêtre, AP-HP, EA7361, Université et Faculté de Médecine Paris-Sud, LabEx LERMIT, Le Kremlin-Bicêtre, France;; b Institut de Chimie des Substances Naturelles, CNRS UPR 2301, Université Paris-Saclay, LabEx LERMIT, Gif-sur-Yvette, France

**Keywords:** Database, beta-lactamase, antibiotic resistance, hydrolytic profile, mutant

## Abstract

Beta-Lactamase Database (BLDB) is a comprehensive, manually curated public resource providing up-to-date structural and functional information focused on this superfamily of enzymes with a great impact on antibiotic resistance. All the enzymes reported and characterised in the literature are presented according to the class (A, B, C and D), family and subfamily to which they belong. All three-dimensional structures of β-lactamases present in the Protein Data Bank are also shown. The characterisation of representative mutants and hydrolytic profiles (kinetics) completes the picture and altogether these four elements constitute the essential foundation for a better understanding of the structure-function relationship within this enzymes family. BLDB can be queried using different protein- and nucleotide-based BLAST searches, which represents a key feature of particular importance in the context of the surveillance of the evolution of the antibiotic resistance. BLDB is available online at http://bldb.eu without any registration and supports all modern browsers.

## Introduction

β-Lactams, due to their safety, reliable killing properties and clinical efficacy, are among the most frequently prescribed antibiotics used to treat bacterial infections. However, their utility is being threatened by the worldwide proliferation of β-lactamases (BLs) with broad hydrolytic capabilities, especially in multi-drug-resistant gram-negative bacteria. These BLs are divided into four classes based on their sequence identities^1^. While a handful of BLs were known in the early 1970s, their number has ever since been growing rapidly, especially with the description in clinical isolates of novel enzymes being capable of hydrolysing carbapenems, last resort antibiotics^2^. A representative example is the class A KPC-2 that in a few years became one of the most menacing BL currently spreading worldwide^3^.

Historically, the principal resource of BLs was maintained from 2001 at the Lahey Clinic (http://www.lahey.org/Studies/) by George Jacoby and Karen Bush, by assigning new enzyme numbers for a number of representative BL families. From July 2015, this resource was transferred into the Bacterial Antimicrobial Resistance Reference Gene Database (https://www.ncbi.nlm.nih.gov/bioproject/313047/) maintained at the NCBI. Other resources are the Institute Pasteur MLST Database (http://bigsdb.pasteur.fr/klebsiella/klebsiella.html), the Antibiotic Resistance Genes Database^4^, the Lactamase Engineering Database^5^^,^^6^, the Metallo-β-Lactamase Engineering Database^7^, the Comprehensive Antibiotic Resistance Database^8^, the β-Lactamase Database^9^, the Comprehensive β-Lactamase Molecular Annotation Resource^10^. However, most of these databases are either not maintained anymore, have a very broad scope or are focused on a few BL families.

The aim of our *Beta-Lactamase Database (BLDB)* is to compile sequence information as well as biochemical and structural data on all the currently known BLs. This comprehensive web-based database, which is updated on a weekly basis, may provide at a glance useful insights in the structure-function relationships of BLs, allowing a better understanding of substrate specificities and key residues involved in substrate recognition and hydrolysis. Altogether, the information provided by BLDB may help to foresee the impact of future mutations on the evolution of BLs.

## Implementation details

The database is hosted on a dedicated virtual server in the cloud, which allows easy adjustments and evolution of computing resources according to the needs.

The core pages are implemented in PHP on a Linux Server under the CentOS 7.2 operating system, whereas the raw data is stored as tabulated files in order to facilitate the updates.

The interactive images showing the list of BL families that are present in the BLDB are generated dynamically in *SVG* format from the raw data, thus ensuring an updated display at any time. The corresponding URL links are directly embedded in the *SVG* images.

Multiple sequence alignments are automatically generated with Clustal Omega^11^ using the default parameters. Phylogenetic trees are processed using Phylip version 3.695 (http://evolution.genetics.washington.edu/phylip/) using Clustal Omega’s *DND* output files and represented as *SVG* images to provide the best quality and minimal file size.

The radar charts representing hydrolytic profiles are dynamically built using a modified and personalised version of the *D3.js* JavaScript library (https://gist.github.com/nbremer/6506614).

The BLAST interface is provided by SequenceServer^12^ and the BLAST + binaries are downloaded from NCBI^13^.

Input data is downloaded from NCBI using the “Entrez Direct: E-utilities on the UNIX Command Line”^13^ and from the PDB with personalised scripts.

Structures are updated semi-automatically on a weekly basis, after each PDB update. New enzymes are added following every update of the Bacterial Antimicrobial Resistance Reference Gene Database. Constant literature survey also provides newly described BLs and synthetic mutants, as well as their hydrolytic profiles. The long-term maintenance of the BLDB is ensured by the collaboration between two academic teams with active interest and experience in the field of BL-mediated antibiotic resistance.

## Database architecture

**Figure 1.**
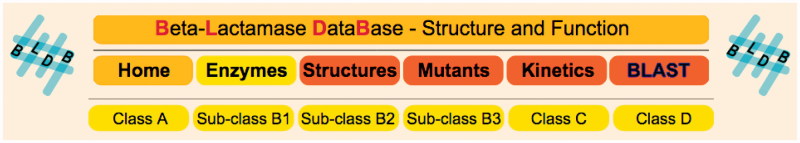
Global architecture of the Beta-Lactamase Database. In addition to the *Home page*, there are five main sections, dedicated to *Enzymes* (classified into the four classes A, B, C and D, and further into the three sub-classes of class B), three-dimensional *Structures* available in the Protein Data Bank, synthetic *Mutants* and hydrolytic profiles (*Kinetics*) described in the literature, and a graphical interface for *BLAST* queries.

BLDB is designed around five main sections, which are strongly interconnected and gathered around the main home page (Figure 1). All pages contain (i) a header showing the overall structure of the BLDB, with links for an easy access to all sections at any moment, and (ii) a footer with acknowledgments to funding bodies that have contributed to this project and with contact details.

### Home page

A short introduction to the present challenges associated with the antibiotic resistance is presented, highlighting the important contribution provided by the BLDB in this field.

Real-time statistics with the number of entries for each type of data present in the BLDB (Enzymes, Structures, Mutants and Kinetics) and for each one of the four classes of BL are also provided. The entries corresponding to the subclasses B1, B2 and B3 of class B are further detailed, for a better presentation of their similarities and differences (Figure S1).

### Enzymes

The Enzymes tab of the main menu gives access to a list of classes and sub-classes of BLs, together with their corresponding BL families (Figure S3). This is represented as an SVG image that is dynamically generated from the raw data, which always ensures up-to-date information.

Each entry of a given BL family contains the class, protein name and eventually alternative names. When the family features several clearly defined sub-families, this information is also present. GenPeptID and GenBankID (with a RefSeq number when provided by NCBI) are also provided, with the corresponding links on the NCBI’s website for more detailed information. Bibliographic data (PubMedID, DOI), functional (phenotype, hydrolytic profile) and genetic (natural or acquired type) information and links to the other sections are also provided (Figure S2).

Sequence alignments are provided for each class, subclass, family and subfamily (Figure S4), together with the corresponding phylogenetic tree (Figure S5).

### Structures

The Structures tab gives access to a table containing all three-dimensional structures of BLs reported in the Protein Data Bank^14^. Each entry contains the name of BL, together with the class or sub-class to which it belongs, followed by the PDB code and resolution (if applicable). The protein sequence is linked to the corresponding UniProt entry and, if appropriate, the existing mutations (extracted from the PDB file content) are shown. Bibliographic data (PubMedID, DOI) allows an easy retrieval of the original articles associated with the structure through links to PubMed and to the journal website. All ligands, buffer molecules and ions present in the structures are highlighted, together with their interaction mode with the protein (non-covalent, covalent, metal coordination). For all these molecules, links to their corresponding dedicated page on the PDB website are provided. Crystallographic details (space group, unit cell parameters, Z-value) are also presented, in order to facilitate the resolution of new structures and to allow an easy comparison of the existing ones (Figure S6).

### Mutants

The synthetic mutants that were described for each enzyme in the literature are presented, together with bibliographical information (PubMedID, DOI) and links to PDB structures and hydrolytic profiles when appropriate (Figure S7). Given the very important number of synthetic mutants described to date, the present version of the BLDB is not complete. More mutants will be added in the near future.

### Kinetics (hydrolytic profiles)

This section is organised in two parts: (i) a table containing the hydrolytic profiles on different β-lactam antibiotics, with values for the turnover number (*k*_cat_), the Michaelis constant (*K_m_*) and the catalytic efficiency (*k*_cat_/*K_m_*) (Figure S8); (ii) a radar chart representing a superposition of hydrolytic profiles selected for easier comparison (Figure S9). The number of hydrolytic profiles currently available in the BLDB is relatively modest, and more entries are scheduled to be added in the near future.

### BLAST

Protein- and nucleotide-based BLAST search capabilities of BLDB are implemented using a personalised version of the SequenceServer graphical interface^12^. The input sequence type (protein or nucleotide) is automatically detected, and the BLAST search type is adapted accordingly. Advanced parameters can be used for the BLAST search in order to obtain more refined results (Figure S10).

The BLAST search is executed using the default parameters and the results are shown using a personalised interface, with the name of BL highlighted in red and links to the corresponding entries on the NCBI’s website. The number of identical residues between the query and each sequence producing a significant alignment is provided, together with the percentage of identity (Figure S11). Together with the E-value, this represents useful information for a quick assessment of the BLAST results. A percentage of 100.00% means that the input sequence is already present in the BLDB, whereas a high sequence identity points out towards the BL class and/or family to which the input sequence might belong.

In the lower part of the results page, the alignments between the query and sequences producing significant alignments are provided (Figure S12).

## Initial content

As of 25 April 2017, BLDB contains 2666 unique enzymes from all four classes of BLs, as well as 810 three-dimensional structures of BLs that are currently available in the Protein Data Bank (PDB)^14^. BLDB also contains 167 mutants and 47 hydrolytic profiles.

## Conclusion

BLDB is developed and maintained by two well-established research groups that are active in the field of BL-mediated antibiotic resistance. This resource is designed to provide appropriate answers to the needs of the research and clinical communities working on antimicrobial resistance.

## Supplementary Material

IENZ_1344235_Supplementary_Material.pdf
