# Supplementary material for: Beta-lactamase database (BLDB) – structure and function
Source: J Enzyme Inhib Med Chem. 2017 Jul 19;32(1):917–9. doi: 10.1080/14756366.2017.1344235 (PMC6445328; doi:10.1080/14756366.2017.1344235)
Supplement: IENZ_1344235_Supplementary_Material.pdf [file IENZ_A_1344235_SM8111.pdf]

Supplementary Information

Beta-Lactamase DataBase (BLDB) – Structure and Function

Thierry Naas[1,*], Saoussen Oueslati[1], Rémy A. Bonnin[1], Maria Laura Dabos[1,2], Agustin Zavala[1,2], Laurent Dortet[1], Pascal Retailleau[2], Bogdan I. Iorga[2,*]

[1] *Service de Bactériologie-Hygiène, Hôpital de Bicêtre, AP-HP, EA7361, Université et Faculté de Médecine Paris-Sud, LabEx LERMIT, Le Kremlin-Bicêtre, France*

[2] *Institut de Chimie des Substances Naturelles, CNRS UPR 2301, Université Paris-Saclay, LabEx LERMIT, Gif-sur-Yvette, France*

*To whom correspondence should be addressed. E-mail: *thierry.naas@aphp.fr* (T.N.), *bogdan.iorga@cnrs.fr* (B.I.I.)

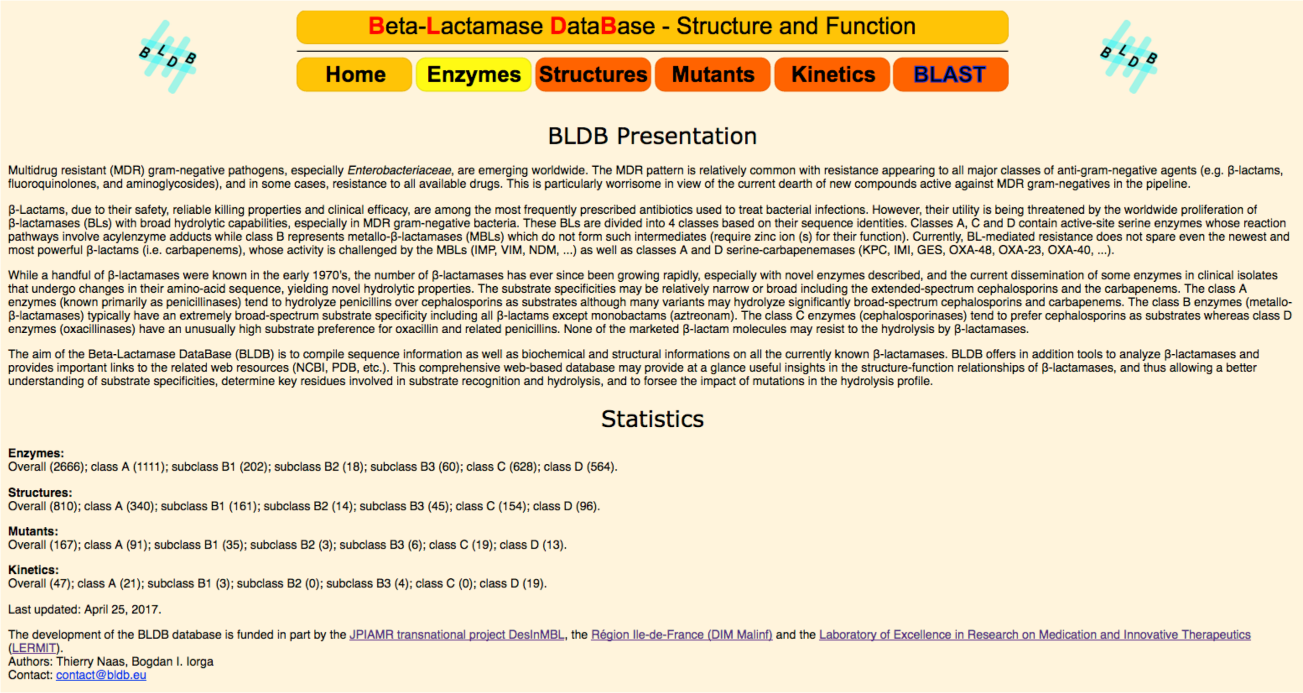

Beta-Lactamase DataBase - Structure and Function

Home | Enzymes | Structures | Mutants | Kinetics | BLAST

BLDB Presentation

Multidrug resistant (MDR) gram-negative pathogens, especially *Enterobacteriaceae*, are emerging worldwide. The MDR pattern is relatively common with resistance appearing to all major classes of anti-gram-negative agents (e.g. β-lactams, fluoroquinolones, and aminoglycosides), and in some cases, resistance to all available drugs. This is particularly worrisome in view of the current dearth of new compounds active against MDR gram-negatives in the pipeline.

β-Lactams, due to their safety, reliable killing properties and clinical efficacy, are among the most frequently prescribed antibiotics used to treat bacterial infections. However, their utility is being threatened by the worldwide proliferation of β-lactamases (BLs) with broad hydrolytic capabilities, especially in MDR gram-negative bacteria. These BLs are divided into 4 classes based on their sequence identities. Classes A, C and D contain active-site serine enzymes whose reaction pathways involve acylenzyme adducts while class B represents metallo-β-lactamases (MBLs) which do not form such intermediates (require zinc ion (s) for their function). Currently, BL-mediated resistance does not spare even the newest and most powerful β-lactams (i.e. carbapenems), whose activity is challenged by the MBLs (IMP, VIM, NDM, ...) as well as classes A and D serine-carbapenemases (KPC, IMI, GES, OXA-48, OXA-23, OXA-40, ...).

While a handful of β-lactamases were known in the early 1970's, the number of β-lactamases has ever since been growing rapidly, especially with novel enzymes described, and the current dissemination of some enzymes in clinical isolates that undergo changes in their amino-acid sequence, yielding novel hydrolytic properties. The substrate specificities may be relatively narrow or broad including the extended-spectrum cephalosporins and the carbapenems. The class A enzymes (known primarily as penicillinases) tend to hydrolyze penicillins over cephalosporins as substrates although many variants may hydrolyze significantly broad-spectrum cephalosporins and carbapenems. The class B enzymes (metallo-β-lactamases) typically have an extremely broad-spectrum substrate specificity including all β-lactams except monobactams (aztreonam). The class C enzymes (cephalosporinases) tend to prefer cephalosporins as substrates whereas class D enzymes (oxacillinases) have an unusually high substrate preference for oxacillin and related penicillins. None of the marketed β-lactam molecules may resist to the hydrolysis by β-lactamases.

The aim of the Beta-Lactamase DataBase (BLDB) is to compile sequence information as well as biochemical and structural informations on all the currently known β-lactamases. BLDB offers in addition tools to analyze β-lactamases and provides important links to the related web resources (NCBI, PDB, etc.). This comprehensive web-based database may provide at a glance useful insights in the structure-function relationships of β-lactamases, and thus allowing a better understanding of substrate specificities, determine key residues involved in substrate recognition and hydrolysis, and to forsee the impact of mutations in the hydrolysis profile.

Statistics

Enzymes:
Overall (2666); class A (1111); subclass B1 (202); subclass B2 (18); subclass B3 (60); class C (628); class D (564).

Structures:
Overall (810); class A (340); subclass B1 (161); subclass B2 (14); subclass B3 (45); class C (154); class D (96).

Mutants:
Overall (167); class A (91); subclass B1 (35); subclass B2 (3); subclass B3 (6); class C (19); class D (13).

Kinetics:
Overall (47); class A (21); subclass B1 (3); subclass B2 (0); subclass B3 (4); class C (0); class D (19).

Last updated: April 25, 2017.

The development of the BLDB database is funded in part by the JPIAMR transnational project DesInMBL, the Région Ile-de-France (DIM Malinf) and the Laboratory of Excellence in Research on Medication and Innovative Therapeutics (LERMIT).
Authors: Thierry Naas, Bogdan I. Iorga
Contact: contact@bldb.eu

Fig. S1. Global overview of the Home page.

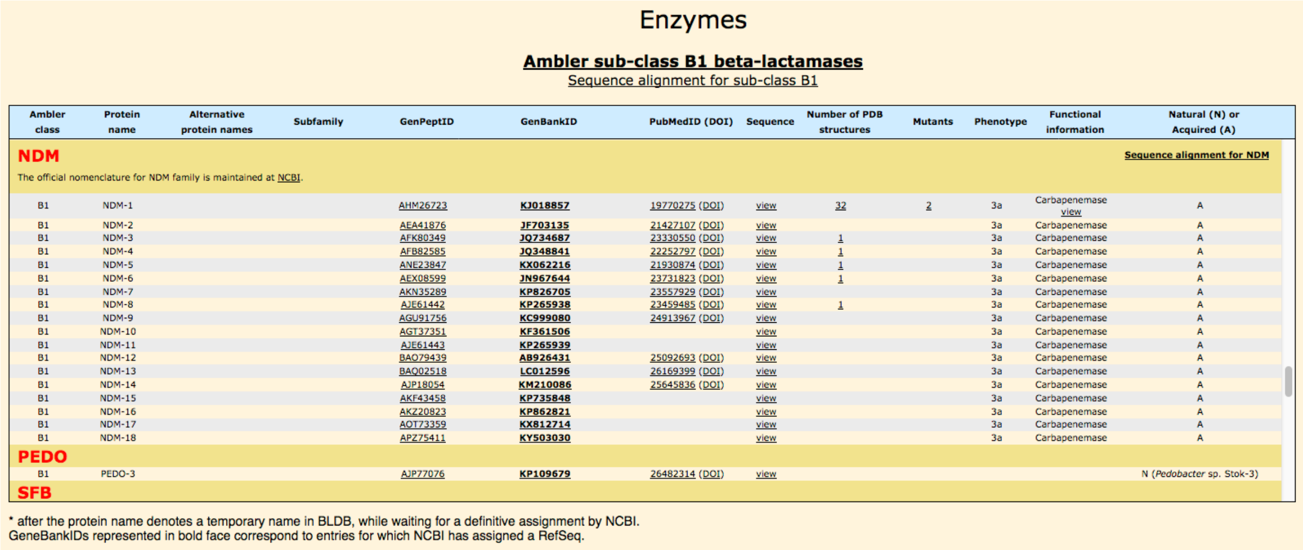

Enzymes

Ambler sub-class B1 beta-lactamases
Sequence alignment for sub-class B1

Ambler class	Protein name	Alternative protein names	Subfamily	GenPeptID	GenBankID	PubMedID (DOI)	Sequence	Number of PDB structures	Mutants	Phenotype	Functional information	Natural (N) or Acquired (A)
**NDM** The official nomenclature for NDM family is maintained at NCBI.												Sequence alignment for NDM
B1	NDM-1			AHM26723	**KJ018857**	19770275 (DOI)	view	32	2	3a	Carbapenemase view	A
B1	NDM-2			AEA41876	**JF703135**	21427107 (DOI)	view			3a	Carbapenemase	A
B1	NDM-3			AFK80349	**JQ734687**	23330550 (DOI)	view	1		3a	Carbapenemase	A
B1	NDM-4			AFB82585	**JQ348841**	22252797 (DOI)	view	1		3a	Carbapenemase	A
B1	NDM-5			ANE23847	**KX062216**	21930874 (DOI)	view	1		3a	Carbapenemase	A
B1	NDM-6			AEX08599	**JN967644**	23731823 (DOI)	view	1		3a	Carbapenemase	A
B1	NDM-7			AKN35289	**KP826705**	23557929 (DOI)	view			3a	Carbapenemase	A
B1	NDM-8			AJE61442	**KP265938**	23459485 (DOI)	view	1		3a	Carbapenemase	A
B1	NDM-9			AGU91756	**KC999080**	24913967 (DOI)	view			3a	Carbapenemase	A
B1	NDM-10			AGT37351	**KF361506**		view			3a	Carbapenemase	A
B1	NDM-11			AJE61443	**KP265939**		view			3a	Carbapenemase	A
B1	NDM-12			BAO79439	**AB926431**	25092693 (DOI)	view			3a	Carbapenemase	A
B1	NDM-13			BAQ02518	**LC012596**	26169399 (DOI)	view			3a	Carbapenemase	A
B1	NDM-14			AJP18054	**KM210086**	25645836 (DOI)	view			3a	Carbapenemase	A
B1	NDM-15			AKF43458	**KP735848**		view			3a	Carbapenemase	A
B1	NDM-16			AKZ20823	**KP862821**		view			3a	Carbapenemase	A
B1	NDM-17			AOT73359	**KX812714**		view			3a	Carbapenemase	A
B1	NDM-18			APZ75411	**KY503030**		view			3a	Carbapenemase	A
**PEDO**												
B1	PEDO-3			AJP77076	**KP109679**	26482314 (DOI)	view					N (*Pedobacter* sp. Stok-3)
**SFB**												

* after the protein name denotes a temporary name in BLDB, while waiting for a definitive assignment by NCBI.
GeneBankIDs represented in bold face correspond to entries for which NCBI has assigned a RefSeq.

Fig. S2. Global overview of the Enzymes section.

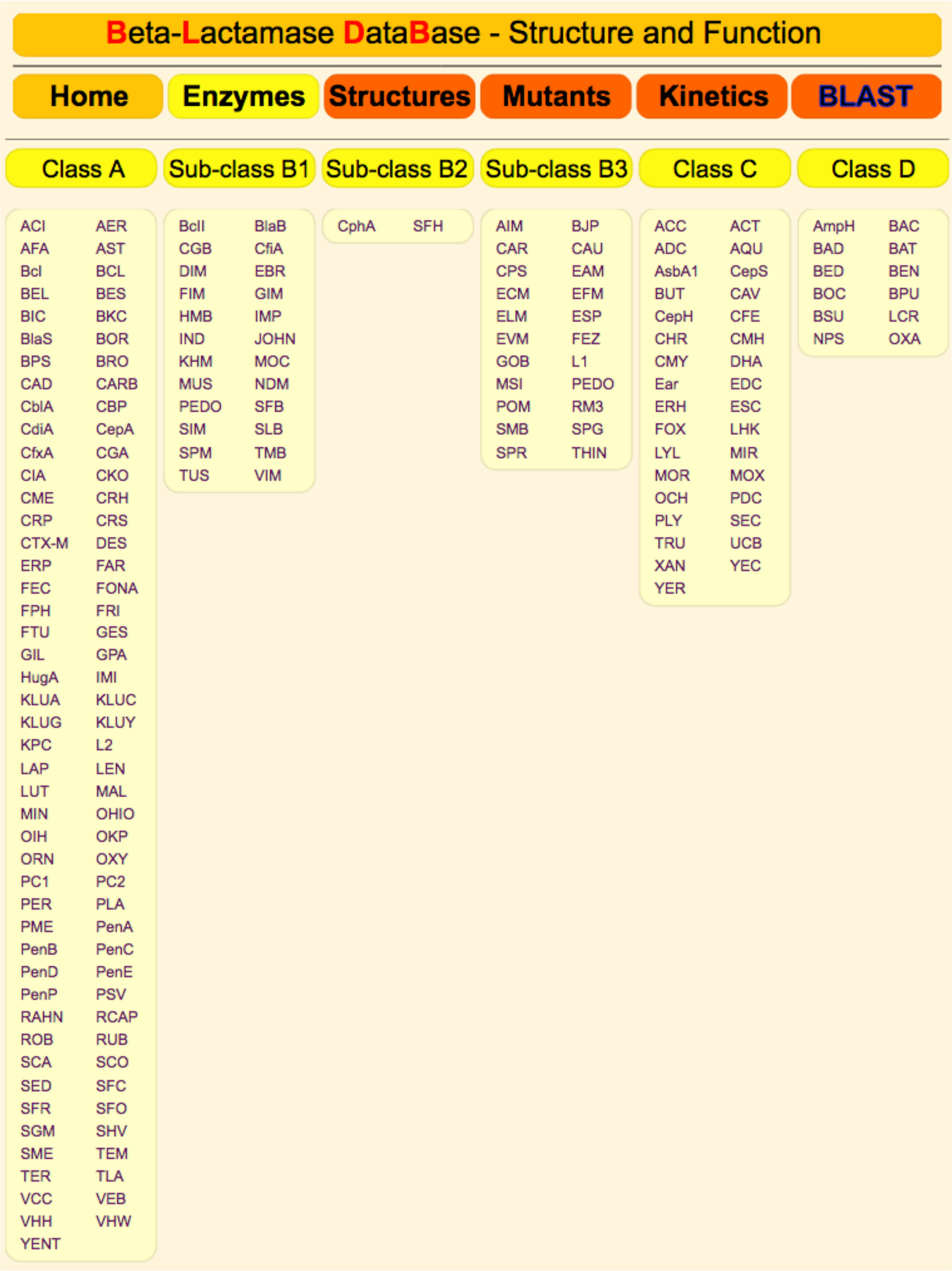

Fig. S3. Global overview of the β-lactamase families that are present in the BLDB.

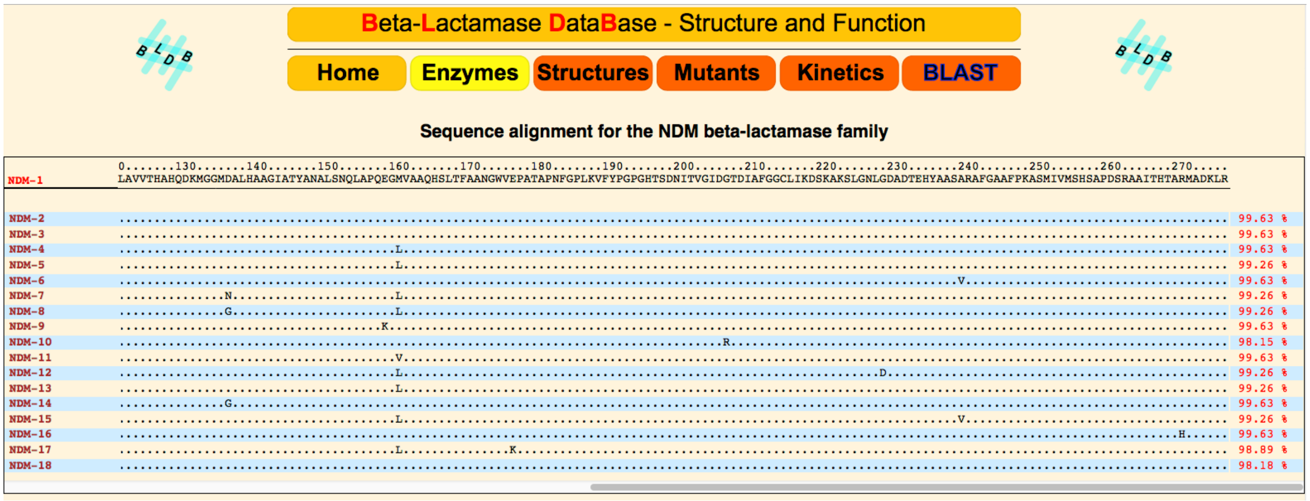

Fig. S4. Sequence alignment.

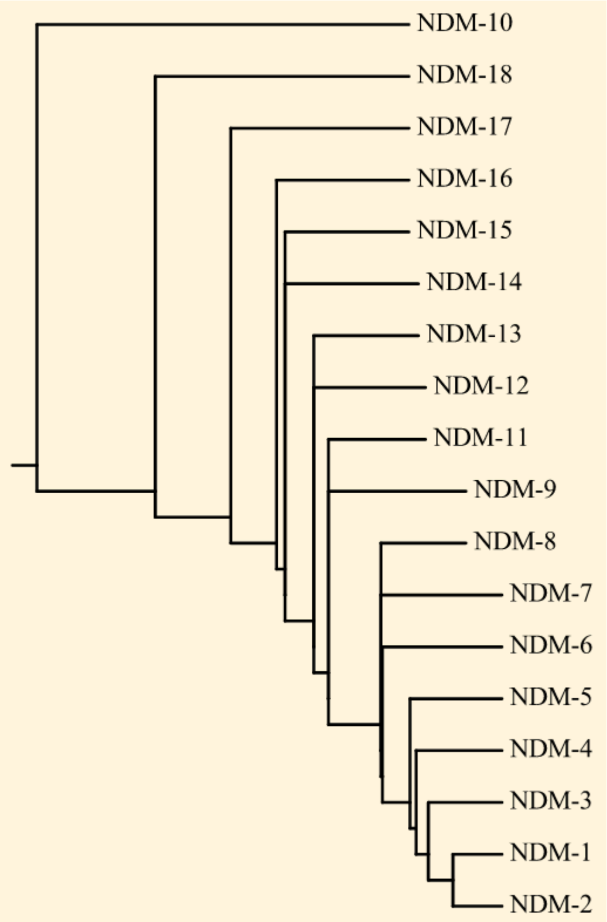

Fig. S5. Rooted phylogenetic tree corresponding to the sequence alignment.

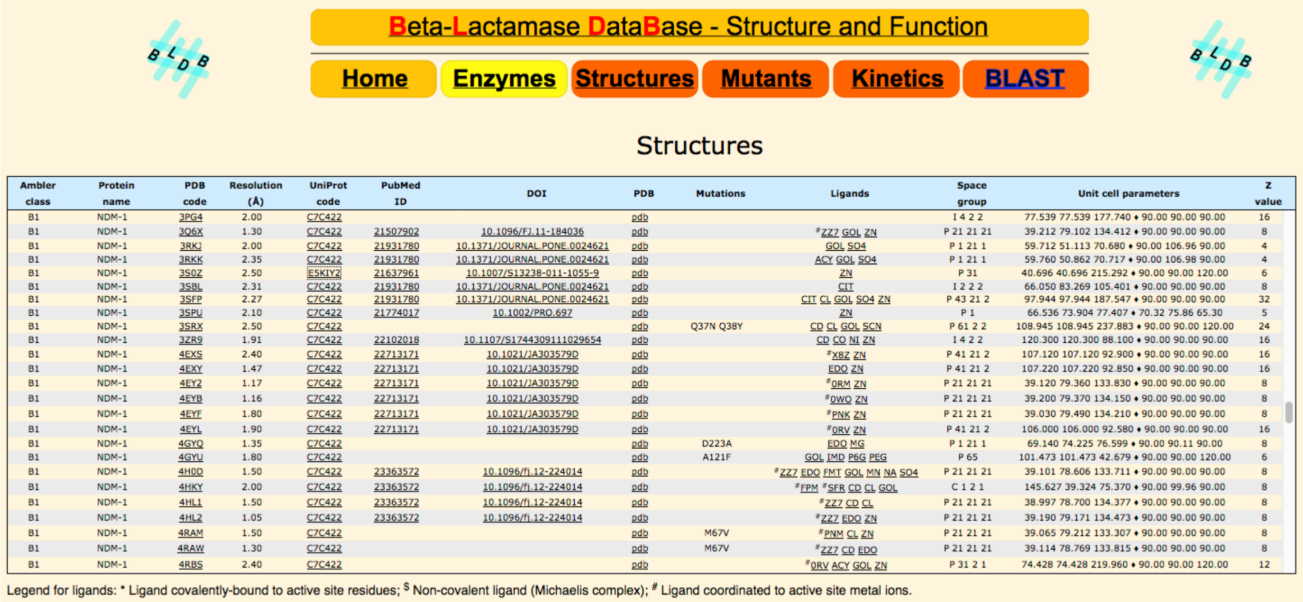

Ambler class	Protein name	PDB code	Resolution (Å)	UniProt code	PubMed ID	DOI	PDB	Mutations	Ligands	Space group	Unit cell parameters	Z value
B1	NDM-1	3PG4	2.00	C7C422			pdb			I 4 2 2	77.539 77.539 177.740 ♦ 90.00 90.00 90.00	16
B1	NDM-1	3Q6X	1.30	C7C422	21507902	10.1096/FJ.11-184036	pdb		#ZZ7 GOL ZN	P 21 21 21	39.212 79.102 134.412 ♦ 90.00 90.00 90.00	8
B1	NDM-1	3RKJ	2.00	C7C422	21931780	10.1371/JOURNAL.PONE.0024621	pdb		GOL SO4	P 1 21 1	59.712 51.113 70.680 ♦ 90.00 106.96 90.00	4
B1	NDM-1	3RKK	2.35	C7C422	21931780	10.1371/JOURNAL.PONE.0024621	pdb		ACY GOL SO4	P 1 21 1	59.760 50.862 70.717 ♦ 90.00 106.98 90.00	4
B1	NDM-1	3S0Z	2.50	E5KIY2	21637961	10.1007/S13238-011-1055-9	pdb		ZN	P 31	40.696 40.696 215.292 ♦ 90.00 90.00 120.00	6
B1	NDM-1	3SBL	2.31	C7C422	21931780	10.1371/JOURNAL.PONE.0024621	pdb		CIT	I 2 2 2	66.050 83.269 105.401 ♦ 90.00 90.00 90.00	8
B1	NDM-1	3SFP	2.27	C7C422	21931780	10.1371/JOURNAL.PONE.0024621	pdb		CIT CL GOL SO4 ZN	P 43 21 2	97.944 97.944 187.547 ♦ 90.00 90.00 90.00	32
B1	NDM-1	3SPU	2.10	C7C422	21774017	10.1002/PRO.697	pdb		ZN	P 1	66.536 73.904 77.407 ♦ 70.32 75.86 65.30	5
B1	NDM-1	3SRX	2.50	C7C422			pdb	Q37N Q38Y	CD CL GOL SCN	P 61 2 2	108.945 108.945 237.883 ♦ 90.00 90.00 120.00	24
B1	NDM-1	3ZR9	1.91	C7C422	22102018	10.1107/S1744309111029654	pdb		CD CO NI ZN	I 4 2 2	120.300 120.300 88.100 ♦ 90.00 90.00 90.00	16
B1	NDM-1	4EXS	2.40	C7C422	22713171	10.1021/JA303579D	pdb		#X8Z ZN	P 41 21 2	107.120 107.120 92.900 ♦ 90.00 90.00 90.00	16
B1	NDM-1	4EXY	1.47	C7C422	22713171	10.1021/JA303579D	pdb		EDO ZN	P 41 21 2	107.220 107.220 92.850 ♦ 90.00 90.00 90.00	16
B1	NDM-1	4EY2	1.17	C7C422	22713171	10.1021/JA303579D	pdb		#0RM ZN	P 21 21 21	39.120 79.360 133.830 ♦ 90.00 90.00 90.00	8
B1	NDM-1	4EYB	1.16	C7C422	22713171	10.1021/JA303579D	pdb		#0WO ZN	P 21 21 21	39.200 79.370 134.150 ♦ 90.00 90.00 90.00	8
B1	NDM-1	4EYF	1.80	C7C422	22713171	10.1021/JA303579D	pdb		#PNK ZN	P 21 21 21	39.030 79.490 134.210 ♦ 90.00 90.00 90.00	8
B1	NDM-1	4EYL	1.90	C7C422	22713171	10.1021/JA303579D	pdb		#0RV ZN	P 41 21 2	106.000 106.000 92.580 ♦ 90.00 90.00 90.00	16
B1	NDM-1	4GYQ	1.35	C7C422			pdb	D223A	EDO MG	P 1 21 1	69.140 74.225 76.599 ♦ 90.00 90.11 90.00	8
B1	NDM-1	4GYU	1.80	C7C422			pdb	A121F	GOL IMD P6G PEG	P 65	101.473 101.473 42.679 ♦ 90.00 90.00 120.00	6
B1	NDM-1	4H0D	1.50	C7C422	23363572	10.1096/fj.12-224014	pdb		#ZZ7 EDO FMT GOL MN NA SO4	P 21 21 21	39.101 78.606 133.711 ♦ 90.00 90.00 90.00	8
B1	NDM-1	4HKY	2.00	C7C422	23363572	10.1096/fj.12-224014	pdb		#FPM #SFR CD CL GOL	C 1 2 1	145.627 39.324 75.370 ♦ 90.00 99.96 90.00	8
B1	NDM-1	4HL1	1.50	C7C422	23363572	10.1096/fj.12-224014	pdb		#ZZ7 CD CL	P 21 21 21	38.997 78.700 134.377 ♦ 90.00 90.00 90.00	8
B1	NDM-1	4HL2	1.05	C7C422	23363572	10.1096/fj.12-224014	pdb		#ZZ7 EDO ZN	P 21 21 21	39.190 79.171 134.473 ♦ 90.00 90.00 90.00	8
B1	NDM-1	4RAM	1.50	C7C422			pdb	M67V	#PNM CL ZN	P 21 21 21	39.065 79.212 133.307 ♦ 90.00 90.00 90.00	8
B1	NDM-1	4RAW	1.30	C7C422			pdb	M67V	#ZZ7 CD EDO	P 21 21 21	39.114 78.769 133.815 ♦ 90.00 90.00 90.00	8
B1	NDM-1	4RBS	2.40	C7C422			pdb		#0RV ACY GOL ZN	P 31 2 1	74.428 74.428 219.960 ♦ 90.00 90.00 120.00	12

Legend for ligands: * Ligand covalently-bound to active site residues; $ Non-covalent ligand (Michaelis complex); # Ligand coordinated to active site metal ions.

Fig. S6. Global overview of the Structures section.

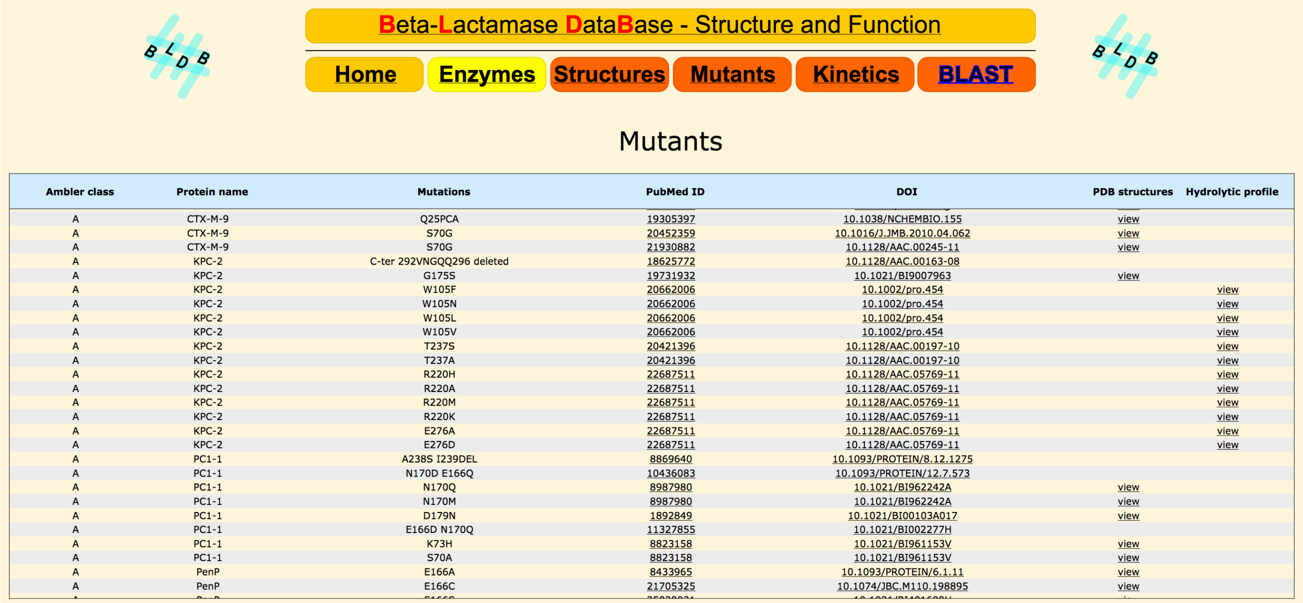

Ambler class	Protein name	Mutations	PubMed ID	DOI	PDB structures	Hydrolytic profile
A	CTX-M-9	Q25PCA	19305397	10.1038/NCHEMBIO.155	view	
A	CTX-M-9	S70G	20452359	10.1016/J.JMB.2010.04.062	view	
A	CTX-M-9	S70G	21930882	10.1128/AAC.00245-11	view	
A	KPC-2	C-ter 292VNGQQ296 deleted	18625772	10.1128/AAC.00163-08		
A	KPC-2	G175S	19731932	10.1021/BI9007963	view	
A	KPC-2	W105F	20662006	10.1002/pro.454		view
A	KPC-2	W105N	20662006	10.1002/pro.454		view
A	KPC-2	W105L	20662006	10.1002/pro.454		view
A	KPC-2	W105V	20662006	10.1002/pro.454		view
A	KPC-2	T237S	20421396	10.1128/AAC.00197-10		view
A	KPC-2	T237A	20421396	10.1128/AAC.00197-10		view
A	KPC-2	R220H	22687511	10.1128/AAC.05769-11		view
A	KPC-2	R220A	22687511	10.1128/AAC.05769-11		view
A	KPC-2	R220M	22687511	10.1128/AAC.05769-11		view
A	KPC-2	R220K	22687511	10.1128/AAC.05769-11		view
A	KPC-2	E276A	22687511	10.1128/AAC.05769-11		view
A	KPC-2	E276D	22687511	10.1128/AAC.05769-11		view
A	PC1-1	A238S I239DEL	8869640	10.1093/PROTEIN/8.12.1275		
A	PC1-1	N170D E166Q	10436083	10.1093/PROTEIN/12.7.573		
A	PC1-1	N170Q	8987980	10.1021/BI962242A	view	
A	PC1-1	N170M	8987980	10.1021/BI962242A	view	
A	PC1-1	D179N	1892849	10.1021/BI00103A017	view	
A	PC1-1	E166D N170Q	11327855	10.1021/BI002277H		
A	PC1-1	K73H	8823158	10.1021/BI961153V	view	
A	PC1-1	S70A	8823158	10.1021/BI961153V	view	
A	PenP	E166A	8433965	10.1093/PROTEIN/6.1.11	view	
A	PenP	E166C	21705325	10.1074/JBC.M110.198895	view	
A	PenP	[illegible]	[illegible]	[illegible]	[illegible]	

Fig. S7. Global overview of the Mutants section.

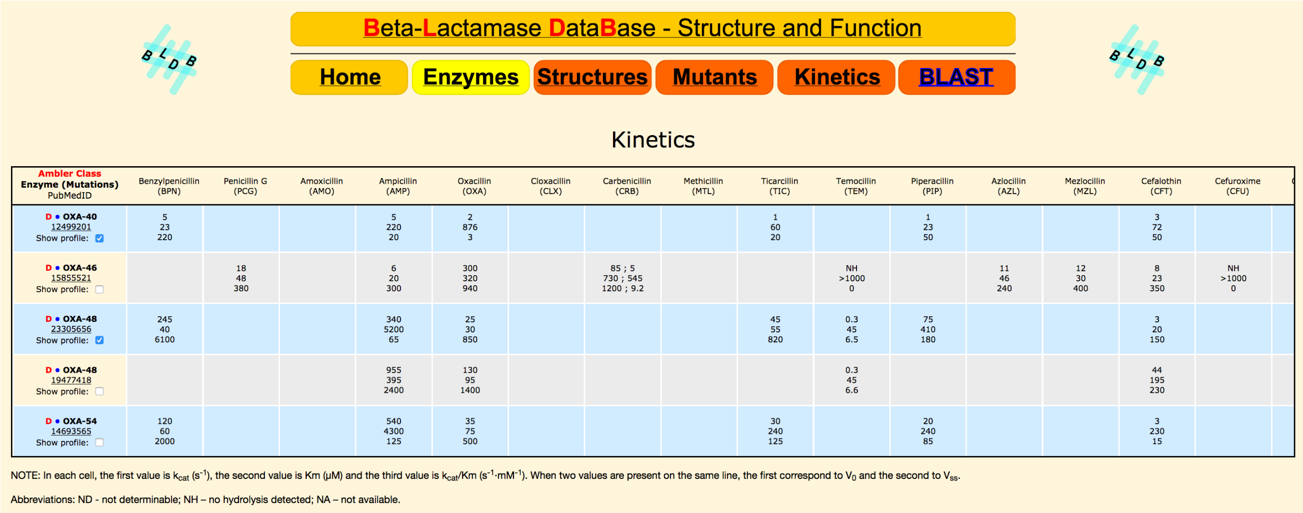

Kinetics

Ambler Class<br>Enzyme (Mutations)<br>PubMedID	Benzylpenicillin (BPN)	Penicillin G (PCG)	Amoxicillin (AMO)	Ampicillin (AMP)	Oxacillin (OXA)	Cloxacillin (CLX)	Carbenicillin (CRB)	Methicillin (MTL)	Ticarcillin (TIC)	Temocillin (TEM)	Piperacillin (PIP)	Azlocillin (AZL)	Mezlocillin (MZL)	Cefalothin (CFT)	Cefuroxime (CFU)
D • OXA-40<br>12499201<br>Show profile: ☑	5<br>23<br>220			5<br>220<br>20	2<br>876<br>3				1<br>60<br>20		1<br>23<br>50			3<br>72<br>50	
D • OXA-46<br>15855521<br>Show profile: ☐		18<br>48<br>380		6<br>20<br>300	300<br>320<br>940		85 ; 5<br>730 ; 545<br>1200 ; 9.2			NH<br>>1000<br>0		11<br>46<br>240	12<br>30<br>400	8<br>23<br>350	NH<br>>1000<br>0
D • OXA-48<br>23305656<br>Show profile: ☑	245<br>40<br>6100			340<br>5200<br>65	25<br>30<br>850				45<br>55<br>820	0.3<br>45<br>6.5	75<br>410<br>180			3<br>20<br>150	
D • OXA-48<br>19477418<br>Show profile: ☐				955<br>395<br>2400	130<br>95<br>1400					0.3<br>45<br>6.6				44<br>195<br>230	
D • OXA-54<br>14693565<br>Show profile: ☐	120<br>60<br>2000			540<br>4300<br>125	35<br>75<br>500				30<br>240<br>125		20<br>240<br>85			3<br>230<br>15	

NOTE: In each cell, the first value is k_{cat} (s^{-1}), the second value is Km (μM) and the third value is k_{cat}/Km ($s^{-1} \cdot mM^{-1}$). When two values are present on the same line, the first correspond to V_0 and the second to V_{ss}.

Abbreviations: ND - not determinable; NH – no hydrolysis detected; NA – not available.

Fig. S8. Global overview of the Kinetics section, showing the hydrolytic profiles.

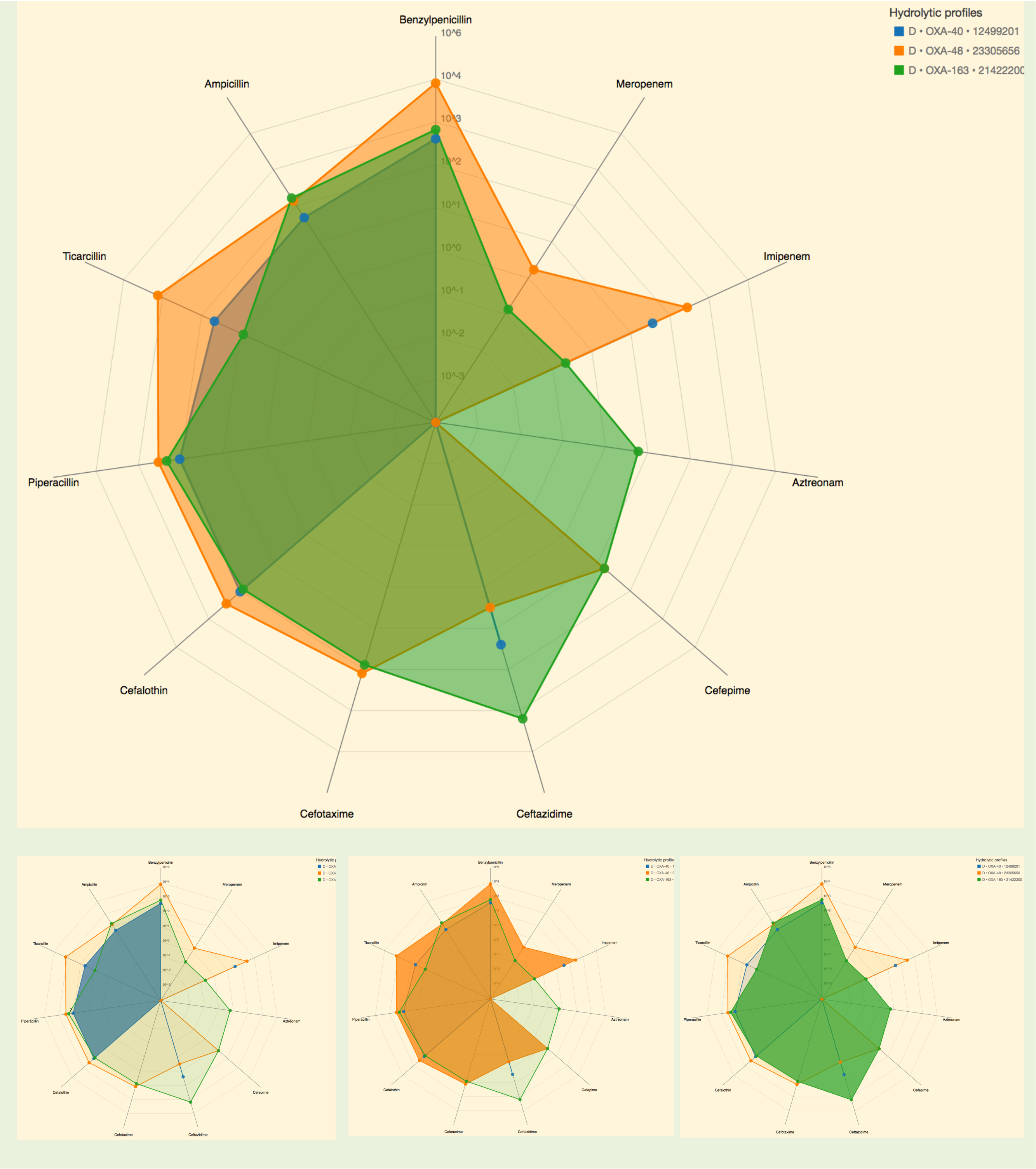

Fig. S9. Radar chart showing the superposition of hydrolytic profiles for OXA-40 (blue), OXA-48 (orange) and OXA-163 (green).

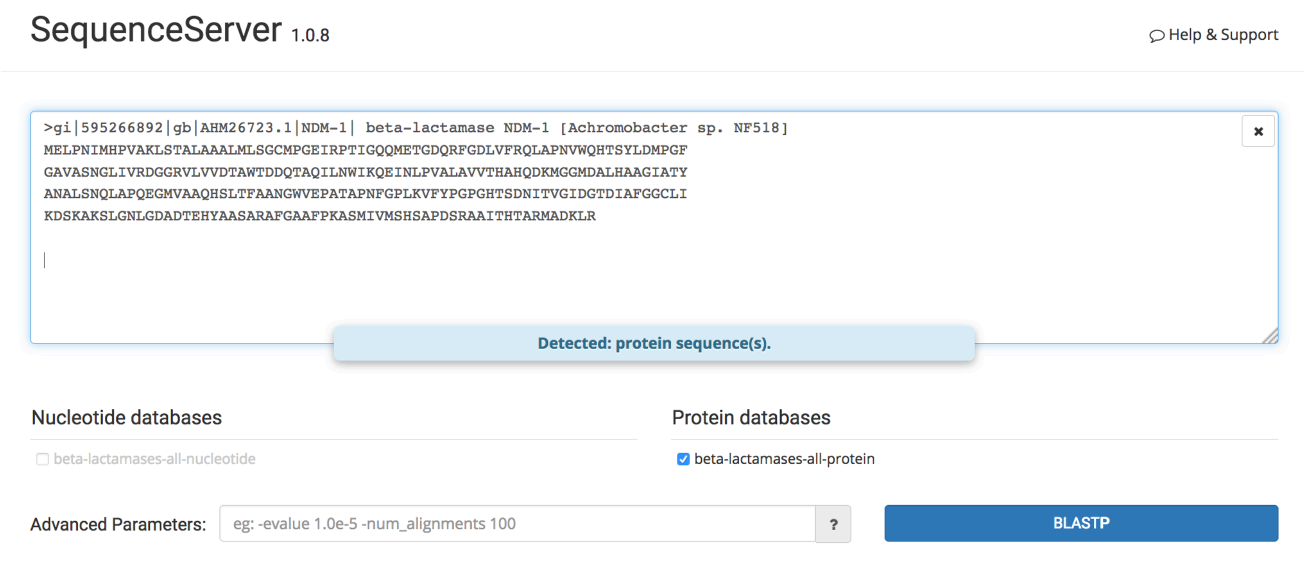

Fig. S10. SequenceServer graphical interface for the nucleotide- and protein-based BLAST queries.

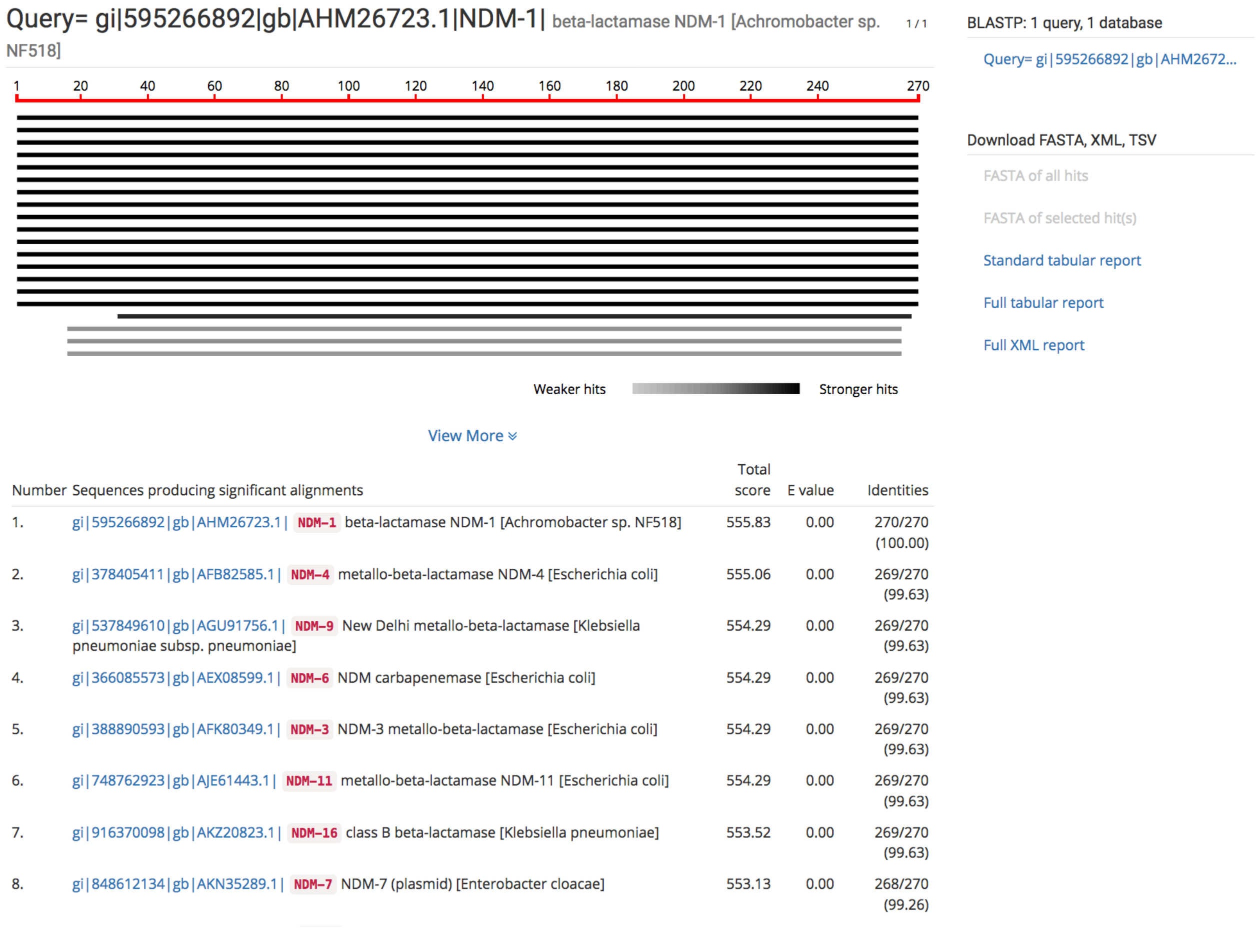

Number	Sequences producing significant alignments	Total score	E value	Identities
1.	gi|595266892|gb|AHM26723.1| NDM-1 beta-lactamase NDM-1 [Achromobacter sp. NF518]	555.83	0.00	270/270 (100.00)
2.	gi|378405411|gb|AFB82585.1| NDM-4 metallo-beta-lactamase NDM-4 [Escherichia coli]	555.06	0.00	269/270 (99.63)
3.	gi|537849610|gb|AGU91756.1| NDM-9 New Delhi metallo-beta-lactamase [Klebsiella pneumoniae subsp. pneumoniae]	554.29	0.00	269/270 (99.63)
4.	gi|366085573|gb|AEX08599.1| NDM-6 NDM carbapenemase [Escherichia coli]	554.29	0.00	269/270 (99.63)
5.	gi|388890593|gb|AFK80349.1| NDM-3 NDM-3 metallo-beta-lactamase [Escherichia coli]	554.29	0.00	269/270 (99.63)
6.	gi|748762923|gb|AJE61443.1| NDM-11 metallo-beta-lactamase NDM-11 [Escherichia coli]	554.29	0.00	269/270 (99.63)
7.	gi|916370098|gb|AKZ20823.1| NDM-16 class B beta-lactamase [Klebsiella pneumoniae]	553.52	0.00	269/270 (99.63)
8.	gi|848612134|gb|AKN35289.1| NDM-7 NDM-7 (plasmid) [Enterobacter cloacae]	553.13	0.00	268/270 (99.26)

Fig. S11. SequenceServer formatted results of a protein BLAST query.

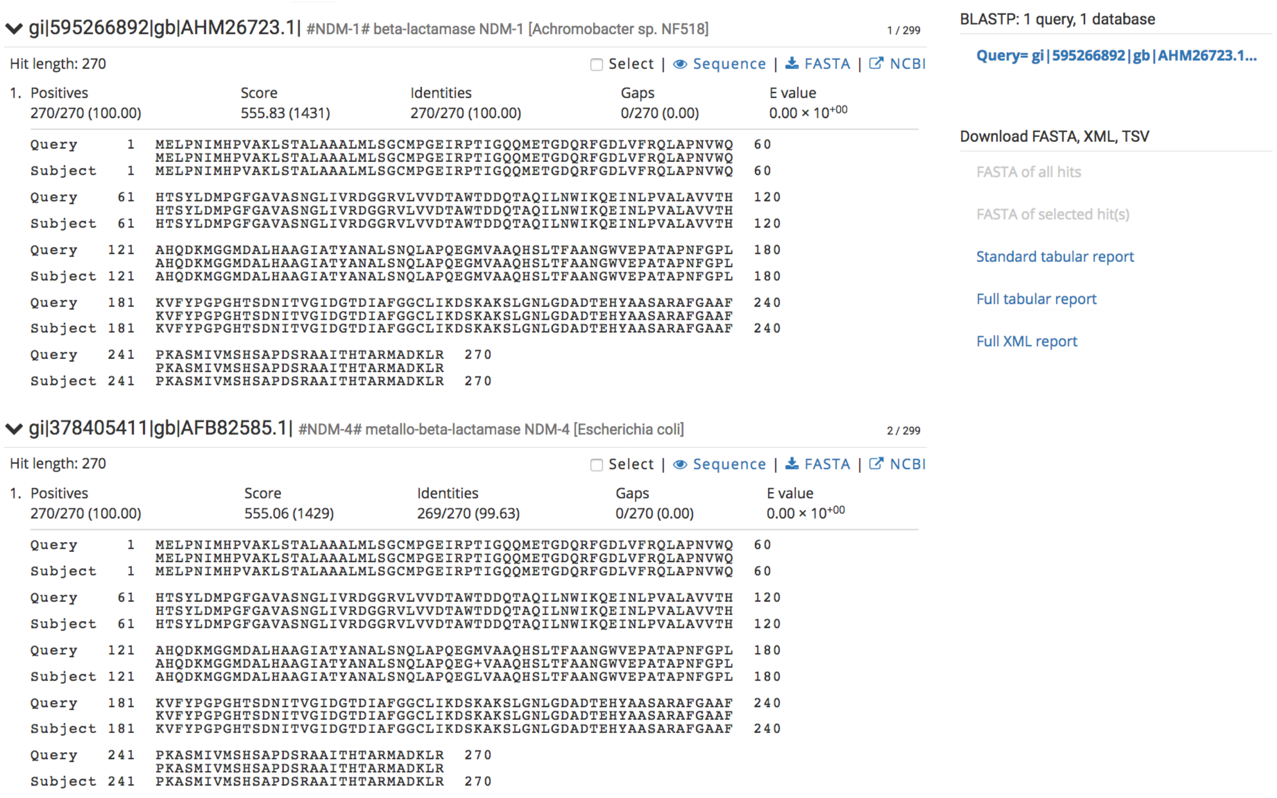

Fig. S12. Sequence alignment of protein BLAST results with the initial query.
